# The Long-Term Clinical Course of Canine Degenerative Myelopathy and Therapeutic Potential of Curcumin

**DOI:** 10.3390/vetsci8090192

**Published:** 2021-09-12

**Authors:** Yui Kobatake, Kohei Nakata, Hiroki Sakai, Jun Sasaki, Osamu Yamato, Satoshi Takashima, Naohito Nishii, Sadatoshi Maeda, Md Shafiqul Islam, Hiroaki Kamishina

**Affiliations:** 1 Faculty of Applied Biological Sciences, Gifu University, 1-1 Yanagido, Gifu 501-1193, Gifu, Japan; shiroki@gifu-u.ac.jp (H.S.); s0takash@gifu-u.ac.jp (S.T.); nishii@gifu-u.ac.jp (N.N.); sadat@gifu-u.ac.jp (S.M.); kamicna@gifu-u.ac.jp (H.K.); 2 The United Graduate School of Veterinary Sciences, Gifu University, 1-1 Yanagido, Gifu 501-1193, Gifu, Japan; k.nakata.1986@gmail.com; 3 Center for Highly Advanced Integration of Nano and Life Sciences, Gifu University, Gifu 501-1193, Gifu, Japan; 4 Faculty of Agriculture, Iwate University, 3-18-8 Ueda, Morioka 020-8550, Iwate, Japan; sasajun@iwate-u.ac.jp; 5 Joint Faculty of Veterinary Medicine, Kagoshima University, 1-21-24 Korimoto, Kagoshima 890-0065, Kagoshima, Japan; osam@vet.kagoshima-u.ac.jp (O.Y.); si.mamun@ymail.com (M.S.I.)

**Keywords:** amyotrophic lateral sclerosis, curcumin, degenerative myelopathy, dogs

## Abstract

Canine degenerative myelopathy (DM), recognized as a spontaneous model of amyotrophic lateral sclerosis, is known as a late-onset progressive degenerative disease of the spinal cord. Because of the progressive nature of DM, many dogs are elected to be euthanized, resulting in limited information on the end-stage clinical presentation. We investigated the long-term clinical course from diagnosis to natural death to further deepen our understanding of the entire clinical picture of this disease. Because curcumin was administered in some cases, the therapeutic effect of curcumin on DM was also examined. Forty dogs included in this study were client-owned Pembroke Welsh Corgis with a definitive diagnosis of DM by necropsy and histopathology. Dogs were excluded from this study if they died from another disease or were elected to be euthanized. Information on the long-term clinical symptoms of DM was investigated based on a questionnaire, which was collected from the dog owners. Urinary incontinence and respiratory disorder were observed in most dogs, as was respiratory impairment-correlated death. In contrast, signs consistent with brainstem dysfunction were noticed at the terminal stage in a small portion of dogs. Although further studies with more cases are needed, the results of this study suggest that administration of curcumin is effective in slowing the progression of DM.

## 1. Introduction

Canine degenerative myelopathy (DM) is a slowly progressive fatal neurodegenerative disorder that affects the spinal cord [1]. A previous study reported that DM was caused by mutations in the gene encoding Cu/Zn superoxide dismutase 1 (SOD1), a key antioxidant enzyme [2]. Further, DM-affected dogs are homozygous for the A allele of a SOD1 missense mutation, SOD1: c.118G>A and c.52A>T, which predicts a p.E40K and T18S amino acid substitution [2,3]. DM has been recently considered a spontaneous model of familial amyotrophic lateral sclerosis (ALS) [4]. ALS is a neurodegenerative disorder characterized by progressive muscle weakness, and 20% of patients with familial ALS have SOD1 gene mutations similar to those found in DM-affected dogs [5,6,7]. In DM and ALS, the exact molecular mechanisms underlying mutant SOD1-mediated neurodegeneration are unclear. Collectively, study results suggest that the cytotoxicity to motor neurons appears to result from a gain of toxic SOD1 function [7,8,9,10], although it has also been proposed that loss-of-function might play a modifying role in ALS [11].

The clinical signs of DM initially appear in the pelvic limbs as spastic upper motor neuron (UMN) paresis and general proprioceptive ataxia, which eventually progresses to flaccid tetraplegia [1]. Furthermore, affected dogs exhibit respiratory muscle disorder and respiratory dysfunction [12,13]. It is important to understand the long-term clinical course of DM in dogs to inform their symptomatic treatment by veterinarians. Understanding the long-term clinical course of DM in dogs could also determine their usefulness as an animal model for ALS in humans. This has not previously been documented in detail, largely because most dogs with DM are elected to be euthanized [14].

Although DM affects many dog breeds [15], an increasing number of Pembroke Welsh Corgis (PWCs) have been affected by this disease in Japan [16]. The increased prevalence of DM in PWCs is attributed to the high SOD1 mutation rate and the large number of animals raised. In addition, PWCs are easier to handle than other predisposed large breed dogs, allowing many owners to provide appropriate care for a longer duration. In the present study, we investigated the long-term clinical course, in particular the incidence and onset of clinical symptoms other than gait abnormality, in 40 DM-affected PWCs from diagnosis to natural death to deepen our understanding of the entire clinical presentation of this disease. In addition, some included cases received curcumin, which is effective for treating ALS [17]. Therefore, the effects of curcumin on DM were also investigated in this study.

## 2. Materials and Methods

### 2.1. Case Recruitment

We recruited dogs with DM through our website on the condition that their owner or attending physician allowed an autopsy of their dog and completed a questionnaire. Our study group included six dogs that attended our hospital after receiving required permissions. Data on these cases were collected from medical records. All the dogs included in this study were client-owned PWCs with a definitive diagnosis of DM by necropsy and histopathology at the Animal Medical Center of the Joint Department of Veterinary Medicine, Gifu University. All dogs were submitted for a necropsy by their owners or their veterinary clinics with the owner’s permission. Histopathological diagnosis was made by veterinary pathologists (HS and JS) using spinal cord sections. For all dogs, SOD1 genotypes were determined by real-time polymerase chain reaction as described in a previous study [16]. For inclusion, cases were required to satisfy the criteria that a neurological clinical manifestation should have been present and the owners should provide responses to a questionnaire detailing their dog’s clinical history. Dogs were excluded from this study if they died from another disease or were elected to be euthanized.

### 2.2. Clinical Symptoms and Treatment

Information was obtained from a questionnaire mainly consisting of items related to clinical symptoms. The following information was obtained via the questionnaire: sex, age of onset, disease duration (from onset to death), and presence and onset of clinical manifestations (Appendix A). Questionnaire data such as the clinical symptoms of the six dogs that visited our hospital were obtained from medical records with the permission of the owners. Eight of the 40 cases had been administered an oral supplement containing curcumin (Neuroact, Veterinarian Medical Development Company Limited, Saitama, Japan). The curcumin dose was approximately 13 mg, which was administered in 1 to 2 divided doses. Their outcomes were compared to the disease progression of cases with no curcumin supplementation. Continuous variables are reported as the median and range.

### 2.3. Statistical Analyses

The Spearman rank-order correlation was used to evaluate the relationship between the onset of dyspnea and survival time. The median age at onset of DM and the onset timing of each symptom were compared across the two treatment groups (curcumin versus control) using the Mann–Whitney U-test. The Kaplan–Meier survival curve, together with a log-rank test, was used to compare the overall survival of the two treatment groups. The confidence interval was set to 95%, and significance was set at *p* < 0.05. All statistical analyses were performed with the statistical software program (EZR, Saitama Medical Center, Jichi Medical University, Saitama, Japan), which is a graphical user interface for a certain software (R, The R Foundation for Statistical Computing, Vienna, Austria).

## 3. Results

### 3.1. Cases

In total, 40 dogs were included in this study. Among them, 26 dogs were male, 21 of which were castrated, and 14 dogs were female, nine of which were spayed. The median age of onset was 10 years and eight months old (range: seven years and seven months to 14 years old). The median age at death was 13 years and nine months old (range: 10 years and three months to 16 years and one month old), and the median survival time (date of symptom onset until date of death) was 36 months (range: 18 to 52 months). All dogs were homozygous for the c.118G>A mutation in the SOD1 gene.

### 3.2. Clinical Symptom

**Figure 1:**
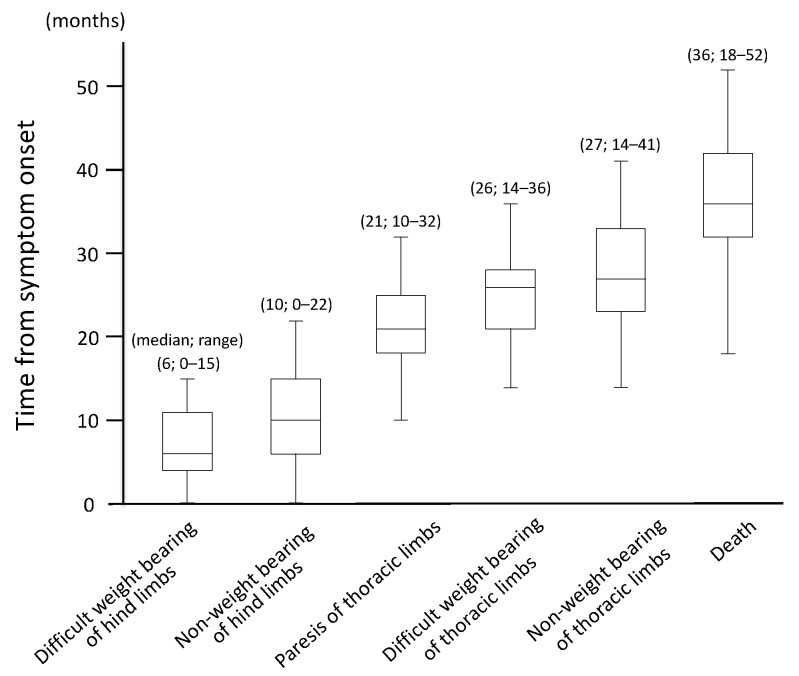
Time course of gait impairment in 40 dogs with degenerative myelopathy.

Without exception, all cases initially presented with progressive generalized proprioceptive ataxia of the pelvic limbs as spastic UMN paresis, which eventually progressed to flaccid tetraplegia. The time course for gait impairment among the 40 dogs with DM is shown in Figure 1. Furthermore, the incidence and onset of symptoms other than gait disturbance is shown in Table 1 and Appendix B. Urinary incontinence was evident in 31 dogs, and 17 of these 31 dogs also showed fecal incontinence. Fecal incontinence was observed simultaneously or subsequent to the onset of urinary incontinence. Eleven dogs showed dysphonia, in particular hoarseness, but the timing of onset differed substantially between individuals. Four cases exhibited dysphagia, and three of four dogs died within two months of the onset of dysphagia; the remaining dog survived for seven months after the onset of dysphagia. Tongue spasms occurred in five dogs. One dog demonstrated a decline in the perception of the face, which suggested trigeminal nerve paralysis. Another dog had loss of facial movement, which indicated facial nerve paralysis. Tongue spasms and facial and trigeminal nerve paralysis arose in the patients who lived longer than the median survival time.

**Figure 2:**
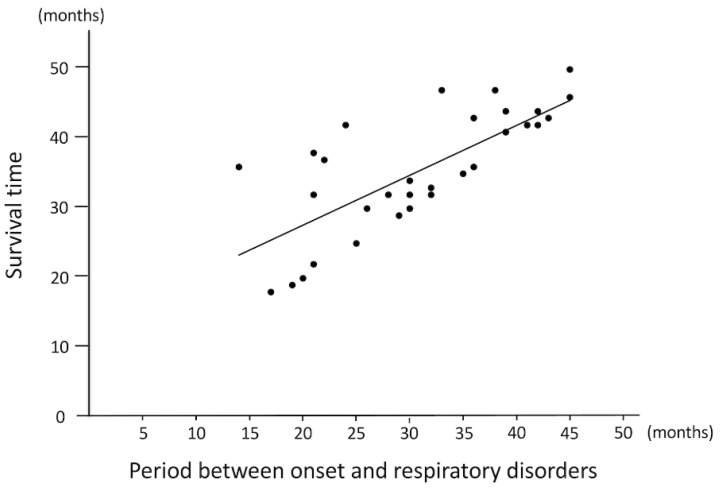
The relationship between the onset of dyspnea and survival time (*n* = 34).

Dyspnea was recognized in a high percentage of the dogs observed (34 of 40 dogs; 85.0%). The median survival time from the onset of dyspnea was 1.5 months (range: 0–22 months), and 11 out of the 34 dogs died within one month of the onset of dyspnea. Meanwhile, five of 34 cases lived more than a year after the onset of dyspnea. The correlation coefficient between the onset of dyspnea and survival time was 0.76 (*p* < 0.0001; Figure 2), suggesting that respiratory impairment was correlated with death. Symptomatic treatment such as oxygen inhalation was provided in these cases when deemed necessary, as judged by their regular veterinarian.

All cases first exhibited progressive paresis of the pelvic limbs, which eventually progressed to flaccid tetraplegia.

These results suggest that the onset of dyspnea is correlated with survival time (R = 0.72, *p* < 0.0001).

### 3.3. Treatment

**Figure 3:**
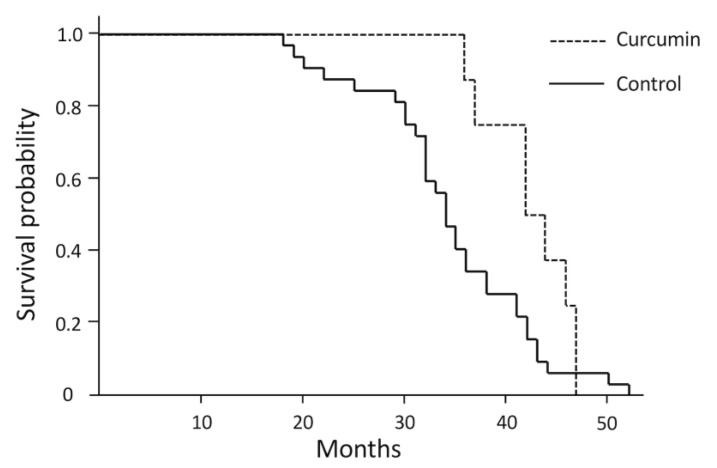
A Kaplan–Meyer survival curve for curcumin-treated and control dogs.

Dogs in the curcumin-treated group included one intact and four neutered males and one intact and two spayed females. No adverse events associated with treatment were observed throughout the study. The dogs treated with curcumin had a significantly longer survival time than those who had not received treatment (Figure 3); Table 2 compares the disease progression of DM in dogs in the curcumin-treated and control groups. The timing of the onset of thoracic limb paresis was not significantly different between the curcumin and control groups; however, nonweight bearing of the hind/thoracic limbs was prolonged in the curcumin-treated dogs when compared with the control dogs. Owing to the small number of cases presenting clinical symptoms other than gait disturbance, analyzing whether there was a difference between the curcumin-treated and control groups was not possible. However, as respiratory disorder was a common symptom, we compared the timing of respiratory disorder onset between the two groups but found no significant difference.

The survival time of the curcumin-treated group (*n* = 8) was significantly longer than that of the control group (*n* = 32; *p* = 0.04 by log-rank test).

## 4. Discussion

Canine DM is a fatal disease, hence almost all dogs in previous studies characterizing its clinical evolution were euthanized [1,14]. Therefore, to date, the median survival time and the later clinical course of DM have not been documented in detail. This study is the first report indicating the natural clinical course of DM. The age of disease onset and sex distribution of PWC dogs included in this study was not notably different from those of previous studies [1,14,15]. One previous study demonstrated that the total duration of clinical signs before euthanasia was 19 months [14], whereas the present study exhibited a median survival time of 36 months.

For all DM cases, symptoms began as hind limb weakness, with the affected dogs eventually developing tetraplegia; the symptoms of patients with ALS tend to vary depending on the type of gene mutation [18]. Dogs with DM have an SOD1 missense mutation (E40K or T18S), and all cases in this study only exhibited an E40K mutation; therefore, all cases may have shown similar symptoms. The clinical progression of DM was different across patients, but the progression duration of gait abnormality was similar to that of a previous description [1].

In dogs, DM leads to dysfunction of the cranial nerve, and urinary and fecal incontinence [1]; however, the timing of onset of these clinical signs has not been characterized. This study indicated that urinary and fecal incontinence was present in a high percentage of dogs with DM, which was similar to what was noted in patients with ALS [19]. The regulation of urination and defecation involves a complex neural control system in the brain, spinal cord, and peripheral autonomic neurons [20]. In dogs with DM, spinal cord lesions spread in ascending and descending orders from the caudal thoracic spinal cord [21], leading to the development of urinary and fecal incontinence. In this study, fecal incontinence had a lower incidence than urinary incontinence. Since the peripheral nervous system of the alimentary tract is more developed and autonomic than that of the urinary tract, automatic peristalsis may have occurred in many dogs with DM. Although this survey did not investigate how care was taken for incontinence, daily compression urination is required for cases with urinary incontinence. In addition, urinary and fecal incontinence induces dermatitis, making it necessary to instruct the owner to keep the skin clean. Furthermore, because a urinary tract infection is likely to occur in cases with incontinence, antibiotic treatment should be considered necessary.

Some dogs with DM presented symptoms of brainstem disorder, such as dysphonia, dysphagia, tongue spasms, and facial and trigeminal nerve paralysis. The nuclei of cranial nerve neurons in the medulla oblongata participate in swallowing, vocalization, movement of the tongue and face, and the sense of touch. As such, we expected that these symptoms would simultaneously develop; however, dysphonia was shown earlier than the other brainstem disorder symptoms. The action of barking requires the movement of the larynx and vibration of the vocal cords by breathing; thus, respiratory dysfunction may have been related to the early expression of dysphonia.

In patients with ALS, death is usually caused by respiratory failure owing to the loss of motor neurons supplying innervation to the diaphragm and chest wall muscles [22]. Moreover, dogs affected by DM are known to exhibit respiratory muscle disorders and respiratory dysfunction [12,13]. Our results demonstrate that respiratory dysfunction occurs in many dogs with DM, and this is the first report to indicate a relationship between the survival time and breathing impairment of dogs with DM.

Various therapeutic protocols have been tested as interventions to slow the disease progression of ALS. Many studies have developed novel therapeutic agents for ALS using rodent models; however, they have had poor therapeutic success when translated to human patients with ALS. It was presumed that the poor translation of the therapies was due to these ALS transgenic animals overexpressing specific human gene mutations [23]. Compared with the SOD1 rodent models, dogs with DM are more similar to ALS patients in terms of the size, structure, and complexity of their nervous systems as well as in terms of the duration of the disease. For these reasons, dogs with DM can be considered a good disease model for the evaluation of potential therapeutic interventions for ALS [4].

We found that dogs with DM treated with curcumin had a significantly longer survival time than those who did not receive this treatment. Curcumin is a biologically potent polyphenolic compound that has various effects on the central nervous system, including neuroprotective effects such as antioxidant and anti-inflammatory activities [24]. Furthermore, a previous study suggested that curcumin molecules bound strongly to the aggregation-prone regions of the mutant SOD1 proteins and blocked the exposed aggregation site, leading to the inhibition of the formation of SOD1 unstructured aggregates [25]. In addition, there are three known beneficial effects of curcumin on muscles: (1) promotion of protein synthesis; (2) reduction of muscle protein degradation; and (3) reduction of exercise muscle injuries [26,27]. There were no statistically significant differences in the duration from onset to the paresis of thoracic limbs in the curcumin and control groups. Nevertheless, the duration from onset to the nonweight bearing of hind/thoracic limbs was longer in dogs from the curcumin group. This suggests that curcumin may be efficient in preventing neurodegeneration and muscle atrophy in dogs with DM. Overall, the results of this study suggest that the administration of curcumin has a neuroprotective and muscle-protective potential against DM, and perhaps against ALS as well. However, dog owners who choose to administer curcumin to their dogs, despite the unproven effectiveness of curcumin in DM, may be more engaged in conservative care such as physical therapy. Therefore, extended survival may be because of the dog owner’s approach to care and not because of the therapeutic effects of curcumin. Furthermore, curcumin possess anti-inflammatory and antioxidant effects on different organs other than the nerves and muscles [28]. Therefore, improvement in motor function may be attributed to the effect of curcumin on joints [29] rather than to the pathology of DM itself.

This study had several limitations. First, the number of available treatment cases was small, thereby weakening the statistical analyses and increasing the chance of errors. Second, this study was retrospective in nature, and there were likely a variety of disease management practices employed by the dog owners. In terms of DM supportive therapy, only daily intensive physiotherapy has demonstrated some benefit, leading to a longer survival and longer maintenance of ambulation when compared to patients who received moderate physiotherapy or no physiotherapy [30]. In our study, the curcumin-treated group may have received more physical therapy and routine care than the control group. For example, aggressive physiotherapy moderates the decline in gait function, and appropriate repositioning for quadriplegic dogs prevents pressure ulcers and reduces the likelihood of aspiration. Similarly, if careful observation by the owner reveals respiratory abnormalities in the affected dog, oxygen replacement therapy can be immediately considered, which leads to a more effective respiratory function. Thus, the response of the owner, rather than curcumin administration, may have prolonged the survival of the curcumin-treated group. Furthermore, the time of onset of DM and time from symptom onset are subjective judgments by the owner and may therefore be affected by the personality of the owner and home environment. Finally, the blood concentration of curcumin was not uniform across dogs with DM, suggesting the possibility of differences in bioavailability. Furthermore, other neuroprotective substances, in addition to curcumin, may have contributed to or protected against the progression of DM. Additional prospective studies, with uniform conditions, are required to confirm the effectiveness of curcumin.

## 5. Conclusions

This study has improved the characterization of the end-stage clinical presentation of DM in dogs, particularly urinary and fecal incontinence, brainstem disorders, and dyspnea. Dyspnea may be related to the death of dogs with DM as well as that of patients with ALS. The characterization of longitudinal changes in the clinical presentation of DM will assist veterinary practitioners and owners in recognizing when and how to provide appropriate care to DM-affected dogs and will be helpful in determining target therapies.

## Figures and Tables

**Table 1:** Prevalence and time from symptom onset other than gait disorder.

Symptoms	Prevalence (%)	Time from Symptom Onset (Range), Month
Respiratory disorder	34/40 (85.0%)	32 (14–45)
Urinary disorder	31/40 (77.5%)	22 (8–38)
Fecal disorder	17/40 (42.5%)	22 (8–37)
Dysphonia	11/40 (27.5%)	31 (19–38)
Tongue spasm	5/40 (12.5%)	42 (35–43)
Dysphagia	4/40 (10.0%)	34 (31–42)
Trigeminal nerve paralysis	1/40 (2.5%)	47
Facial nerve paralysis	1/40 (2.5%)	39

**Table 2:** Comparison of disease progression of degenerative myelopathy between curcumin-treated and control dogs.

Time from Symptom Onset	Curcumin (Range), Months	Control (Range), Months	*p*-Value
Age at onset of clinical symptom(s)	127 (105–153)	128.5 (91–168)	0.74
Difficult weight bearing of hind limbs	11.5 (8–15)	5.5 (0–15)	0.02
Nonweight bearing of hind limbs	15 (7–18)	8 (0–22)	0.02
Paresis of thoracic limbs	22.5 (16–30)	21 (10–37)	0.27
Difficult weight bearing of thoracic limbs	30 (27–33)	24 (14–36)	0.004
Nonweight bearing of thoracic limbs	34.5 (24–41)	26 (14–40)	0.007
Respiratory failure	35.5 (14–45)	31 (17–45)	0.73
Survival time	43 (36–47)	34 (18–52)	-

## Data Availability

All datasets obtained and analyzed during the experiment are available upon reasonable request from the respective author.

## Appendix A

The content of the questionnaire.

1. Profile of the case
  - Age at onset
  - Disease duration (from onset to death)
  - Sex
2. Dysfunction of gait
  - The time from symptom onset to difficult weight bearing of hind limbs
  - The time from symptom onset to nonweight bearing of hind limbs
  - The time from symptom onset to paresis of thoracic limbs
  - The time from symptom onset to difficult weight bearing of thoracic limbs
  - The time from symptom onset to nonweight bearing of thoracic limbs
3. The other abnormality
  - The time from symptom onset to urinary disorder
  - The time from symptom onset to fecal disorder
  - The time from symptom onset to respiratory disorder
  - Other clinical symptoms (If your dog has other symptoms, please provide details of the symptoms and the time from symptom onset.)
4. Survival time
  - The time from symptom onset to death
5. Treatment (If your dog was being treated, please fill this out).

## Appendix B

**Table A1:** Details of clinical symptoms and treatment for each case.

No	Survival Time (Month)	Age at Death	Symptoms Other Than Gait Disorder	Administration of Curcumin
1	25	15 years 6 months	Respiratory, Urinary, Fecal	
2	50	14 years 2 months	Respiratory	
3	34	14 years	Urinary, Fecal	
4	22	14 years	Respiratory,	
5	32	10 years 3 months	Respiratory, Urinary	
6	33	14 years 11 months	Respiratory, Dysphonia	
7	42	15 years 9 months	Respiratory, Urinary, Fecal	
8	30	14 years 6 months	Respiratory, Urinary	
9	52	15 years 9 months	Urinary	
10	42	12 years 3 months	Respiratory	
11	34	13 years	Respiratory, Urinary, Fecal	
12	34	13 years 6 months	Urinary, Dysphagia	
13	31	12 years 7 months	Urinary	
14	36	15 years 4 months	Respiratory, Dysphonia	
15	42	12 years 3 months	Respiratory, Urinary, Fecal, Dysphonia	curcumin
16	36	13 years 4 months	Respiratory, Urinary, Fecal, Dysphagia	
17	32	15 years	Respiratory, Urinary, Dysphonia	
18	19	15 years 7 months	Respiratory	
19	32	14 years 10 months	Respiratory, Urinary	
20	38	14 years 4 months	Urinary, Fecal	
21	43	13 years 6 months	Respiratory	
22	41	14 years 6 months	Respiratory, Urinary, Fecal, Facial nerve	
23	20	13 years 2 months	Respiratory	
24	44	12 years 1 month	Respiratory, Urinary, Fecal	
25	43	14 years 4 months	Respiratory, Urinary	
26	29	12 years 1 month	Respiratory, Urinary, Fecal	
27	41	12 years 9 months	Respiratory, Urinary, Fecal, Dysphonia	
28	30	14 years 4 months	Respiratory, Urinary, Dysphonia	
29	36	13 years 3 months	Respiratory, Urinary	curcumin
30	32	13 years 1 month	Respiratory, Urinary, Fecal	
31	35	13 years 1 month	Respiratory, Urinary	
32	35	12 years 6 months	Urinary, Fecal	
33	18	12 years	Respiratory, Urinary	
34	38	12 years 8 months	Respiratory, Urinary, Fecal, Dysphagia	
35	41	16 years 8 months	Respiratory, Urinary, Fecal, tongue	curcumin
36	37	15 years 10 months	Respiratory, Urinary, Fecal, Dysphonia	curcumin
37	47	13 years 6 months	Respiratory, Urinary, Fecal, Dysphonia, Tongue, Trigeminal nerve	curcumin
38	46	16 years 1 month	Respiratory, Urinary, Tongue, Dysphonia	curcumin
39	42	13 years 5 months	Respiratory, Urinary, Dysphonia, Tongue	curcumin
40	44	14 years 8 months	Respiratory, Dysphonia, Tongue, Dysphagia	curcumin
